# Instance Transfer Subject-Dependent Strategy for Motor Imagery Signal Classification Using Deep Convolutional Neural Networks

**DOI:** 10.1155/2020/1683013

**Published:** 2020-08-28

**Authors:** Kai Zhang, Guanghua Xu, Longtin Chen, Peiyuan Tian, ChengCheng Han, Sicong Zhang, Nan Duan

**Affiliations:** ^1^School of Mechanical Engineering, Xi'an Jiaotong University, Xi'an 710049, China; ^2^State Key Laboratory for Manufacturing Systems Engineering, Xi'an Jiaotong University, Xi'an, China

## Abstract

In the process of brain-computer interface (BCI), variations across sessions/subjects result in differences in the properties of potential of the brain. This issue may lead to variations in feature distribution of electroencephalogram (EEG) across subjects, which greatly reduces the generalization ability of a classifier. Although subject-dependent (SD) strategy provides a promising way to solve the problem of personalized classification, it cannot achieve expected performance due to the limitation of the amount of data especially for a deep neural network (DNN) classification model. Herein, we propose an instance transfer subject-independent (ITSD) framework combined with a convolutional neural network (CNN) to improve the classification accuracy of the model during motor imagery (MI) task. The proposed framework consists of the following steps. Firstly, an instance transfer learning based on the perceptive Hash algorithm is proposed to measure similarity of spectrogram EEG signals between different subjects. Then, we develop a CNN to decode these signals after instance transfer learning. Next, the performance of classifications by different training strategies (subject-independent- (SI-) CNN, SD-CNN, and ITSD-CNN) are compared. To verify the effectiveness of the algorithm, we evaluate it on the dataset of BCI competition IV-2b. Experiments show that the instance transfer learning can achieve positive instance transfer using a CNN classification model. Among the three different training strategies, the average classification accuracy of ITSD-CNN can achieve 94.7 ± 2.6 and obtain obvious improvement compared with a contrast model (*p* < 0.01). Compared with other methods proposed in previous research, the framework of ITSD-CNN outperforms the state-of-the-art classification methods with a mean kappa value of 0.664.

## 1. Introduction

A brain-computer interface (BCI) is a communication method between a user and a computer that does not rely on the normal neural pathways of the brain and muscles. Motor imagery (MI), one of the paradigms of BCI, is a way of thinking that imitates the motor intention without real motion output; that is, the brain imagines the entire movement without contracting the muscles [1]. Research has shown that motor imagery (MI) can produce the same change of sensory motor rhythms as a real movement [2]. This phenomenon will cause energy increase or decrease in specific frequency bands of EEG, which are called event-related desynchronization (ERD) and event-related synchrony (ERS) [3]. The differences of ERD/ERS are always used to decode mental intentions, control a robot, and execute rehabilitation training for stroke patients [4]. During this process, the accurate decoding of MI is the essential factor that determines the effectiveness and quality of the rehabilitation. However, due to the differences in physiological structure and physiological condition across subjects/trials, there will be obvious variations in feature distribution for EEG signals. Especially, as a spontaneous potential activity, the signal of MI is extremely weak and always accompanied with nonlinearity and nonstationary. It brings a huge challenge for the decoding model for MI.

**Figure 1:**
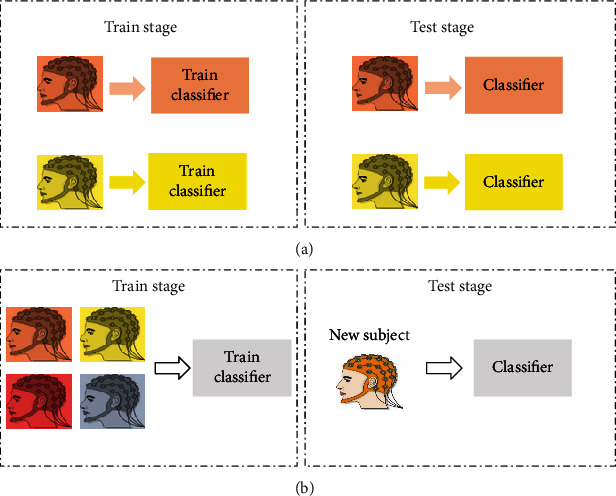
A diagram representing the (a) subject-dependent (SD) and (b) subject -independent (SI) training strategy.

With the development of machine learning (ML) and deep learning (DL) technology, more classification models are widely used for EEG decoding [5]. During the training stage of the classification model, strategy can be divided into two ways: subject-dependent (SD) and subject-independent (SI). As shown in Figure 1, SD strategy is aimed at training a subject-specific model using their own data. In contrast, SI strategy utilizes data from other subjects to train a generalized decoding model for a new subject [6]. One of the main assumptions of ML and DL is that training data and test data belong to the same feature space and subject to the same probability distribution. But it is often violated in the field of EEG signal processing. In other words, the SI strategy cannot satisfy performance of accuracy and generalization due to the individual differences across subject/sessions. SD strategy provides an effective way to optimize this issue; however, it requires long calibration sessions to collect the high-quality and large amounts of EEG datasets. All these restrictions greatly affect the application of BCI in practice.

One effective approach to solve this problem is instance transfer learning (ITL) [7], which combined the advantages of training strategy of SI and SD, i.e., training personalization classification model with enough data. The definition of transfer learning is that given a source domain *D*_*s*_ and learning task *T*_*s*_ and a target domain *D*_*T*_ and learning task *T*_*T*_ transfer learning are aimed at helping improve the performance of target predictive function *f*_*T*_(·) using the knowledge from *D*_*s*_. ITL is one of the typical TL methods, which transfer instance knowledge by reweight the data from *D*_*s*_ to improve generalization ability for *f*_*T*_(·).

**Figure 2:**
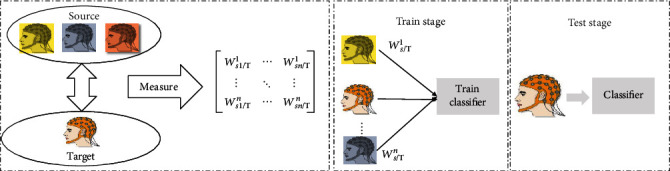
A diagram representing the instance transfer subject-dependent (ITSD) training strategy.

The essence of ITL does not change the feature space or property of signals in MI task, but it finds the optimal transfer weighting coefficient for source data by similarity measurement [8–10]. The transfer weighting coefficient is then weighted with the number of corresponding data from *D*_*s*_. As shown in Figure 2, *w*_*S*_*i*_/*T*_^*k*^ represents the transfer weighting coefficient for *D*_*s*_ data. *k* represents the serial number of the subject, and *i* is the *i*th trials. During the training stage, weighted data from *D*_*s*_ were combined with *D*_*T*_ data to train classifier. Based on this, we could utilize similar EEG data from other subjects or sessions to help reduce system calibration time and train decoding model for target subject [11]. For example, Azab et al. proposed a weighted transfer learning for improving MI task that they use Kullback–Leibler divergence to measure the similarity between two feature spaces of signal. According to the results of similarity, the weight coefficient is assigned to the source data to optimize the small sample problem in classification model training [12]. A Jensen-Shannon ratio method is used to measure similarity between target data with source data in Giles et al.'s work [13]. Based on this method, they propose a target subject identification framework based on rule adaptation transfer learning, which can reduce the calibration time of the online BCI system by reusing the data with the highest similarity between *D*_*s*_ and *D*_*T*_.

However, due to the obvious individual differences across subjects, the direct instance transfer method may bring negative transfer effects. In addition, traditional measurement does not concentrate on the specific feature of EEG data, which will affect the performance of the transfer learning. Especially for motor imagery signals, the traditional time-series signals cannot effectively reflect the feature of motor intention, but the energy feature of signal can represent the distribution of feature well. Therefore, choose the translocatable objects and assign transfer weights reasonably to the core research for instance transfer learning [14]. In the computer vision field, content-based image retrieval (CBIR) is an important research topic [15]. The goal of CBIR is to find images from the source domain that belongs to the same category. MI spectrogram image contains abundant information of frequency and energy feature, which is suitable for extracting feature of motor intention. Therefore, we assume that technology of CBIR may implement effective data matching across subject and achieve the effective instance transfer from *D*_*s*_ to *D*_*T*_. The perceptive Hash (p-Hash) algorithm is one of the typical CBIR methods, which is used to judge the similarity between different images by transforming these images to perceptual hash code and measure its distance [16].

With the development of deep neural network (DNN) technology, EEG decoding based on DNN has attracted wide attention. Due to the excellent ability of fitting and automatic feature extraction, DNNs achieve outperformed results for EEG classification. In Reference [17], a convolutional neural network (CNN) and variational autoencoder (VAE) were used for two-class MI classification task. The CNN utilized multiple hidden layers to extract the features, and the VAE was used for feature classification. The CNN-VAE method achieved a 3% improvement in classification accuracy than the best methods in their referred literature. Lu et al. [18] proposed a novel method based on restricted Boltzmann machines (RBMS) for EEG classification. Fast Fourier transform (FFT) and wavelet packet decomposition (WPD) were used to extract the frequency-domain features of signals, which were used as inputs of the network. Three RBMs were stacked with an additional output layer to train the classification network. The authors verified that the classification performance of this network was better than state-of-art methods evaluated by public datasets (*p* < 0.01). In a recent study [19], the researchers compared the classification performance of a CNN and long short-term memory (LSTM) network for classifying the time-frequency domain signals of MI. The authors evaluated the adaptability between different network structures to individual differences and showed that CNN provided better performance for detecting the differences across subjects, and its classification rate was significantly higher than that of the LSTM. In summary, CNN shows satisfactory classification performance in MI-BCI task compared to traditional machine learning methods or other networks. However, the limitation of the amount of dataset hinders the practical applications of DNN. Especially for SD training strategy, it is difficult for a subject to collect enough high-quality EEG data. Therefore, we propose a novel instance transfer learning based on p-Hash to improve the utilization efficiency of data and build a CNN for MI classification.

Based on the problems mentioned above, we propose a novel instance transfer learning strategy combined with CNN for subject-dependent MI classification. The main contributions of this paper are as follows:

1. To address the issue of individual differences across subjects/sessions in MI classification, we creatively apply the methodology of content-based image retrieval to EEG classification. Based on this, we proposed a novel instance transfer learning (ITL) strategy using the p-Hash algorithm, which is aimed at calculating the transfer weight coefficient between the trails from different subjects/sessions
2. There are two main limitations of subject-dependent and subject-independent training strategies: small-scale dataset and large difference of signal across subjects. Therefore, we apply instance transfer learning to optimize the traditional training strategies. Similarity measurement in feature space is executed to calculate the transfer weight coefficient across subjects/sessions, which is aimed at exploring the correlation between different trials. And then we expand the training set for target subject based on instance transfer by weighted
3. To improve the classification performance in MI-BCI task, we combine CNN with transfer learning strategy using SD training strategy (ITSD-CNN) to classify MI signal. Experiments evaluate that the ITSD-CNN can achieve outperformed results than state-of-art methods

The step of ITSD-CNN can divide into these steps: firstly, we preprocess the raw MI-EEG signals and adopt short-time Fourier transform (STFT) to transform the raw MI signal into a 2-D spectrogram signal. Then, an ITL based on the perceptive Hash algorithm is proposed to measure the similarity of MI signals between *D*_*s*_ and *D*_*T*_. Next, we build a convolutional neural network to classify the MI data after transfer learning. The BCI competition IV-2b dataset is used to verify the effectiveness of this framework. Our results show that the proposed approach can significantly improve the classification performance. Meanwhile, the ITSD provides a new training strategy to optimize the performance of SD training. The rest of the sections is organized as follows.  Section 2 explains the materials and methods for ITSD and CNN.  Section 3 introduces the experimental results and discussion. Discussion is described in Section 4, and Section 5 is the conclusion of the paper.

## 2. Materials and Methods

### 2.1. Description of Datasets

**Figure 3:**
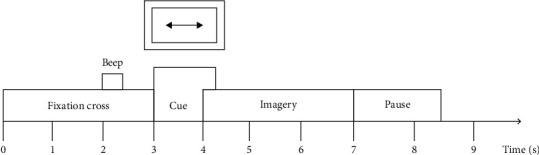
Diagram of a trial and timings.

In this paper, we utilize BCI competition IV dataset 2b [20]. This dataset was provided by the BCI Research Institute in Berlin and contained two parts: the standard set and the evaluation set. Nine subjects participated this experiment, and three channels (C3, C4, and CZ) were used to record EEG with a 250 Hz sampling rate. Each subject is required to imagine the movement of left and right hands according to the cue. And all of them underwent 5-session experiments. The experimental process is shown in Figure 3.

### 2.2. Preprocessing of EEG

MI signals are extremely weak and accompanied with irrelevant component. And the feature of MI tends to appear in specific frequency band: mu band (8-14 Hz) and beta band (14-30 Hz). To reduce the effect of the artifact of signals, we uniformly filter the signal to 8-30 Hz through the Butterworth filter with 6 orders.

**Figure 4:**
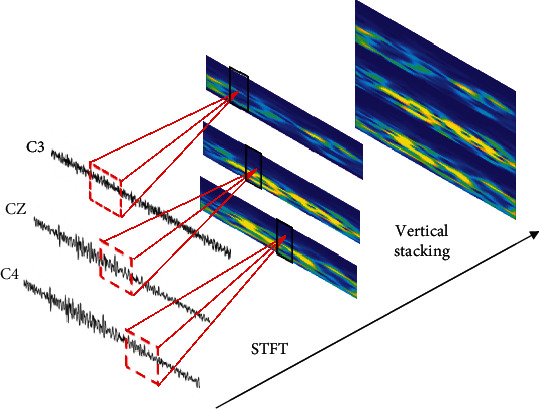
Spectrogram images with 3 electrodes after STFT.

The potential activity of MI always causes the variation of energy in the contralateral cortex and ipsilateral cortex during MI, which is recorded by C3, Cz, C4, and surrounding channels [3]. However, this phenomenon cannot reflect in time domain clearly. To describe features in a better form, we transform time-series signals to spectrogram signals after filtering. As shown in Figure 4, the three channels are converted into a two-dimensional form and are mosaicked into an image using vertical stacking. The variations along *X*-axis and *Y*-axis represent the trend of time series and frequency, respectively. And the depth of color indicates the energy feature. For one trial, we chose the data from 3 to 7 s (period of imagery) and set the window size of the STFT as 256. After transformation, all spectrogram images were resized to 64 × 64 for convenience and consistency in the subsequent calculation.

### 2.3. Instance Transfer Learning Based on the Perceptive Hash Algorithm

The spectrogram signal of MI can vividly reflect the feature variations especially for the energy of frequency band. The perceptive hash (p-Hash) algorithm can obtain the most sensitive information by discrete cosine transform (DCT) in the human and machine vision system [16]. This transformation concentrates the energy on the main diagonal of the image matrix and has effectively removed redundant and irrelevant components. Under specific EEG task, the feature distribution of signals across subjects may exist difference but the form of feature is consistent. Therefore, we assume that change between different modes for MI can be effectively perceived and recognized by p-Hash.

This paper uses p-Hash to measure the similarity of spectrogram data across subject. Then, the obtained similarity is transformed into the ITL coefficient that inputs into a classifier combined with corresponding data. The implementation of ITL based on p-Hash are as follows.

Firstly, some denotation of symbols should be explained. We define *D*_*T*_ representing the target subject's data and *D*_*S*_ is other subjects' data. Let us define *G*_*n*_^*i*^ = {*g*_*t*_^*i*^}_*t*=1_^*n*^ ∈ *D*_*s*_^*l*×*l*^ which is a set of single-trial EEGs represented by spectrogram from *D*_*S*_, *Q*_*n*_^*i*^ = {*q*_*t*_^*i*^}_*t*=1_^*n*^ ∈ *D*_*t*_^*l*×*l*^ for target subject, where *t* is the number of EEG trials, *l* is the dimensions of a square matrix, *i* represents the *i*-th subject. ${\tilde{q}}_{n}^{i}\left(1/n\right)\sum_{t=1}^{n}q_{t}^{i}{\tilde{q}}_{t}$ represent the average spectrogram for the current target subject.

Before calculation, spectrogram images from *D*_*S*_ and *D*_*T*_ are separately resized to 64 × 64 and converted to grayscale level. Then, discrete cosine transform (DCT) is utilized to compress an image:

(1) $$\begin{array}{c} G_{u,u}=\frac{1}{4}\alpha {\left(u\right)}^{2}\overset{n-1}{\underset{x=0}{\sum }}\overset{n-1}{\underset{x=0}{\sum }}g_{x,x}\cos{\left[\frac{\left(2x+1\right)u\pi }{2N}\right]}^{2},\\ Q_{v,v}=\frac{1}{4}\alpha {\left(v\right)}^{2}\overset{n-1}{\underset{y=0}{\sum }}\overset{n-1}{\underset{y=0}{\sum }}{\tilde{q}}_{siy,y}\cos{\left[\frac{\left(2y+1\right)v\pi }{2N}\right]}^{2},\\ \alpha \left(u\right)=\alpha \left(v\right)=\sqrt{\frac{1}{N}},\;u,v=0,\\ \alpha \left(u\right)=\alpha \left(v\right)=\sqrt{\frac{2}{N}},\;u,v\neq 0, \end{array}$$

*α*(*u*) and *α*(*v*) are coefficient matrixes after transformation. *G*_*u*,*u*_ and *Q*_*v*,*v*_ are results after transformation. The energy variations of the image after DCT are mainly concentrated in the low-frequency part [21]. Therefore, the 8 × 8 matrix *d* located in left diagonal is extracted for subsequent calculations. Next, the mean value of DCT coefficients is calculated, respectively:

(2) $$\begin{array}{c} m_{s}=\frac{1}{n\times n}\overset{n}{\underset{u=1}{\sum }}\overset{n}{\underset{u=1}{\sum }}d_{u,u},\\ m_{t}=\frac{1}{n\times n}\overset{n}{\underset{v=1}{\sum }}\overset{Nn}{\underset{v=1}{\sum }}d_{v,v}. \end{array}$$

In addition, the mean value of DCT coefficients is set as threshold standard to compare with each coefficient. By the rule of threshold, the two-dimensional matrix of *n* × *n* can be compressed into one dimension of 1 × *n* matrix *H*_*i*_.

(3) $$\begin{array}{c} h_{i}=0,\;b_{i}<m,\\ h_{i}=1,\;b_{i}>m, \end{array}$$

where *h*_*i*_ is the bit of the perceptual hash at position *i*, *m* is the mean value of the DCT coefficients, and *b*_*i*_(*i* = 0, 1, ⋯, *N* − 1) is DCT coefficient of the array. The obtained 1 × *n* matrix is *H*_*i*_ which represents perceptual hash code [22].

Finally, respectively, calculate the Hamming distance *d*_*H*_ of perceptual hash code from *D*_*T*_ and *D*_*S*_ and set the distance *d*_*H*_(*H*_*T*_, *H*_*S*_) as the ITL weight coefficient from source domain to target domain. For each trial from source subject, weight *w*_*S*_*i*_/*T*_*t*__ can be calculated:

(4) $$\begin{array}{c} w_{S_{i}/T_{t}}=d_{H}\left(H_{T},H_{S}\right)=\overset{L}{\underset{i=1}{\sum }}{\left[{\text{H}}_{T}\left(i\right)-H_{S}\left(t\right)\right]}^{2}. \end{array}$$

**Figure 5:**
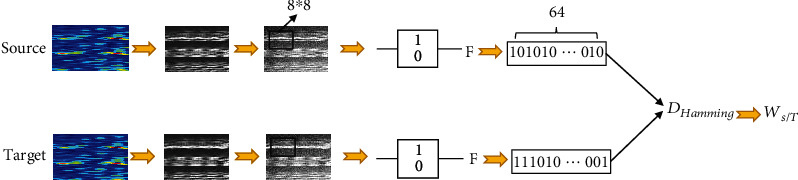
Transfer weight calculation using perceptive hash algorithm.

The calculation processing of transfer weight is shown in Figure 5.

### 2.4. Convolutional Neural Network

Researches show that the CNN has obvious advantages in processing MI signals [23]. CNN is a multilayer neural network composed of a sequence of convolution, pooling, and fully connected layers. The convolution layer extracts different levels of feature of input image by kennel size, while the pooling layer reduces the complexity of the model by subsampling. With the increase of layers, the more advanced features can be extracted. The fully connected layer will transform the output matrix from the last layer to a *n*-dimensional vector (*n* is the number of classes). Backpropagation is utilized to decrease the classification error.

In the convolution layer, the input image can be convolved with a spatial filter to form the feature map and output function, which is expressed as

(5) $$\begin{array}{c} {\text{X}}_{j}^{l}=f\left(\underset{i\in M_{j}}{\sum }X_{i}^{l-1}\times w_{ij}^{l}+b_{j}^{l}\right). \end{array}$$

This formula describes the *j*th feature map in layer l. *X*_*j*_^*l*^ is calculated by the previous feature map *X*_*i*_^*l*−1^ multiplied by the convolution kernel *W*_*ij*_^*l*^ and bias parameter *b*_*j*_^*l*^. Finally, the mapping is completed by RELU function *f*().

(6) $$\begin{array}{c} f\left(a\right)=\text{RELU}\left(a\right)=\ln\left(1+e^{a}\right). \end{array}$$

The pooling layer is sandwiched in the continuous convolution layer to compress the amount of data and parameters and reduce overfitting. The max pooling method in this work is chosen as follows:

(7) $$\begin{array}{c} X_{j,k}^{l}=\max_{0\leq m,n\leq s}\left(X_{j\cdot s+m,k\cdot s+n}^{l-1}\right), \end{array}$$

where *j* and *k* are the locations of the current feature map*X*_*j*_^*l*^ and *s* stands for pooling size. The double fully connected layer structure can effectively translate the multiscale features of the image. Considering the multiple influencing factors of time, frequency, and channel, this paper uses double full-connection layers to improve the performance gain of the softmax layer. Two-way softmax in the last layer in the deep networks is used to predict the distribution of two motor imagery tasks.

(8) $$\begin{array}{c} y_{i}=\frac{\exp\left(\sum x_{i}\cdot w_{i,j}+b_{j}\right)}{\sum \exp\left(\sum x_{i}\cdot w_{i,j}+b_{j}\right)}. \end{array}$$

*x*_*i*_ is the *i*th feature map and *y*_*i*_ represents an output probability distribution. The gradient of back-propagation was calculated according to the cross-entropy loss function.

(9) $$\begin{array}{c} \text{Loss}=-\left[y\log\tilde{y}+\left(1-y\right)\log\left(1-\tilde{y}\right)\right]. \end{array}$$

**Figure 6:**
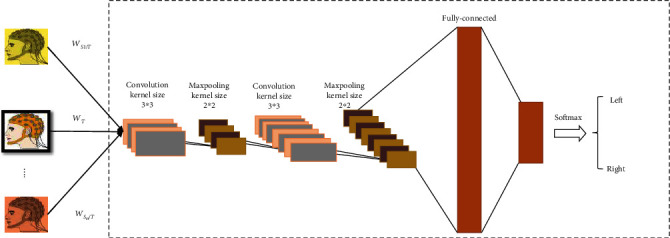
The structure of convolutional neural network for classification.

And we used the stochastic gradient descent (SGD) optimizer with a learning rate of 1*e* − 4 to improve the speed of network training.

(10) $$\begin{array}{c} W^{k}=W^{k}-\mu \frac{\text{tial}E}{\text{tial}W^{k}},\\ b_{k}=b_{k}-\mu \frac{\text{tial}E}{\text{tial}b_{k}}, \end{array}$$

where *μ* is the learning rate, while *W*^*k*^ represents the weight matrix for kernel k and *b*_*k*_ represents the bias value. *E* represents the difference between desired output and real output. There are eight layers in the proposed network (Figure 6).

The first layer is the input layer, and the second layer is a convolutional layer with kernel size 3 × 3; the next layer is the max pooling layer with kernel size 2 × 2. The next two layers have the same kernel size and function. Two fully connected layers, respectively, consist of 10 and 2 neuros to compute the predicted labels. The gradient of backpropagation is calculated according to the cross-entropy loss function. The stochastic gradient descent with momentum (SGDM) optimizer is used for optimization with learn rate = 1*e* − 4, momentum = 0.9, and decay = 1*e* − 6. We set the minibatch size to 50 and the max epoch to 6. To reduce computation time and prevent overfitting, we adopt to the dropout operation. The proposed CNN model is summarized in Table 1.

### 2.5. Evaluation for Classification Performance

In our study, the average classification accuracy and mean kappa value are utilized to test the performance of the proposed framework. The kappa value is a typical method to evaluate the EEG classification performance which can remove the effect of result of random classification. It can be calculated as follows:

(11) $$\begin{array}{c} \text{kappa}=\frac{\text{acc}-\mathrm{rand}}{1-\mathrm{rand}} \end{array}$$

**Figure 7:**
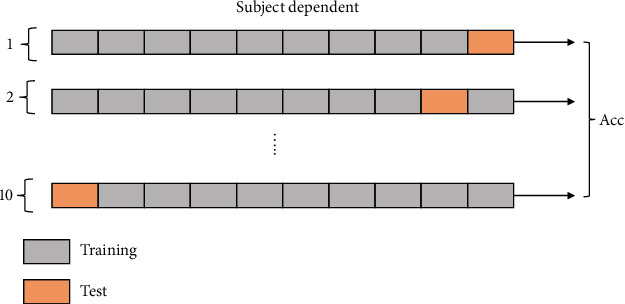
Subject-dependent training strategy.

To evaluate the effectiveness of instance transfer learning, three training strategies are compared. As for the subject dependent method (Figure 7), a total of 720 trials for one subject are divided into the training data and test data using10-fold cross-validation.

**Figure 8:**
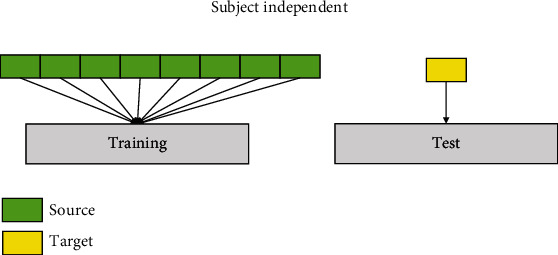
Subject-independent training strategy.

A generalized model is trained using data from other subjects (*D*_*s*_) in the subject-independent training stage (Figure 8).

**Figure 9:**
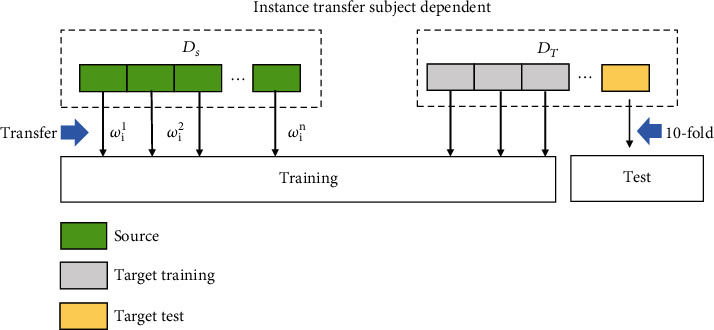
Instance transfer subject-dependent training strategy.

In the ITSD method, weighted data from *D*_*s*_ together with target data are input into the training set. And data from *D*_*T*_ are used to test model performance; the method of data partition is shown in Figure 9.

To show the size of training and test data more clearly, we briefly summarize the number of data for three training strategy in Table 2.

## 3. Experimental Results and Analysis

### 3.1. Performance of the Proposed Framework

In this paper, we use BCI competition IV dataset 2b to verify the proposed methods. During the training stage for each subject in ITSD, we supply dataset from other subjects by instance transfer (Figure 9). After the mixture of source data and target data, we adjust the number of instances to keep the class balance in the training stage. To evaluate the performance of different training strategies, we compared the classification accuracy of different methods.

As depicted in Table 3, the SD training strategy is better than SI based on the CNN classifier even though SI obtains more training data. This indicates that MI-EEG from different subjects causes an obvious difference of feature under the same label. The average classification accuracy of ITSD-CNN is superior to that of SD-CNN, which obtains a 14.1% improvement. It is worth noting that subjects 2 and 3 can better adapt to model preference by efficiently data transfer to greatly improve the classification accuracy.

**Figure 10:**
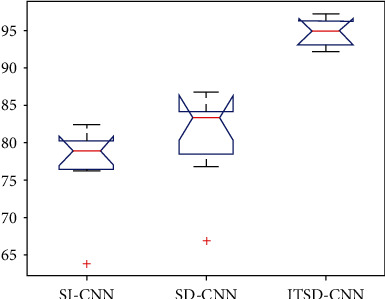
The ANOVA stats of classification accuracy for the compared model.

To verify the significance of results, analysis of variance was performed. As shown in Figure 10, there is no significant difference between SD-CNN and SI-CNN, while the strategy of ITSD-CNN performs satisfied convergence and high accuracy than the other two methods (*p* < 0.01).

By observing the training process, the weakness of small sample can directly influence the results of network training. Effective data transfer can increase the number of samples to improve the generalization of network and prevent underfitting. Moreover, this method can validly reduce the influence of classifier result from individual differences.

### 3.2. Comparisons with Previous Research

**Figure 11:**
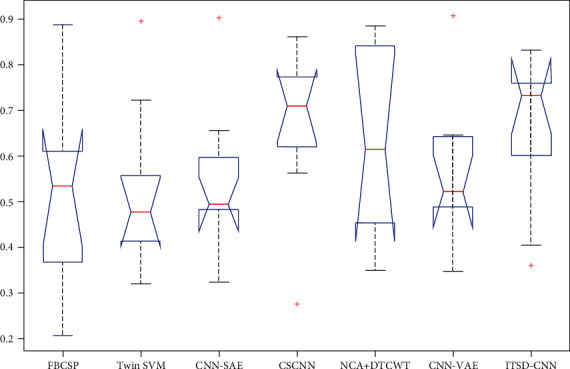
The ANOVA stats of the mean kappa for existing methods.

Numbers methods have been proposed for MI classification using BCI competition IV dataset 2b. In this section, we further compared our method with that of a commonly used strategy by the metric of mean kappa value. Based on the analysis of Table 4 and Figure 11, we can observe that ITSD-CNN outperforms the baseline and the state-of-the-art methods. It is worth noting that the hybrid framework based on CNN obtains an ideal result among these methods. This indicates that CNN has strong robustness and high accuracy in MI classification. In addition, instance transfer effectively improves the classification performance of CNN using the same model and parameters.

## 4. Discussion

Compared with traditional methods, the application of deep learning for EEG classification has successfully improved the performance [28]. However, there are still some limitation hinder its application in practice. The feature distribution of EEGs always shows a difference in the same mental task across subject/session, which may cause the overfitting during the network training. Transfer learning turns out to be instrumental in subject/session classification performance. It can be used to initialize a BCI using knowledge transfer from other subjects for a naive subject. At the same time, this strategy may help a classifier to learn global features from all subjects without falling into the local optimal. Therefore, transfer learning combines the advantages of SI and SD strategy and outperforms them. In future studies, it is valuable to design a decoding model for EEGs based on transfer learning combined with the deep neural network.

Another limitation is small-scale sample for classifier training. Strict requirements of quality and collection of EEG data make it difficult to obtain large datasets in practice. The performance of EEG decoding based on DNN is directly related to the amount of training data. Data augmentation is a promising way to address this issue. As discussed in References [29, 30], artificially generated data can be used to training classification model and the augmentation method has been proved efficient in EEG decoding. The addition of generated dataset improves the complexity and robustness of models. Traditional augmentation methods contain geometric transformation and model generation, which requires a long time to prepare and select suitable generated data. It takes up a lot of computing resources in the BCI system. Therefore, data augmentation from an available database may provide a probable method. As proposed in this study, instance transfer learning can easily obtain data from other subjects and adaptively assign weights to the transfer data, which achieve the utilization maximization of data across subjects. Although the training process of this method is similar with subject-dependent strategy, i.e., it requires recomputing for a new participant; low-cost calculation would not burden the operation of the BCI system. In later research, we will explore the detail of variability across subjects and achieve more effective transfer learning.

## 5. Conclusion

In this paper, we propose a novel instance transfer learning method with a deep neural network applying for the subject-dependent classification of motor imagery in the BCI system. In this work, we firstly transform the raw data to spectrogram image by STFT. Then, instance transfer learning based on the perceptive Hash algorithm is utilized to measure the similarity between the data of source domain and target domain. Next, we convert the similarity into a transfer weight coefficient to realize the data transfer of a single trial between different subjects. Finally, a convolutional neural network is built to verify the performance of proposed methods and some other methods are adopted to evaluate the results. Experiment verifies that instance transfer learning by the perceptive Hash algorithm can effectively provide data augmentation based on subject-dependent training strategy and improve the performance of the classifier, which demonstrates the superior performance and promising potential of proposed novel training strategy. Meanwhile, the proposed method provides a solution for the weakness of small samples in the deep neural network.

## Figures and Tables

**Table 1:** Detailed architecture for the CNN.

Layers	Type	Size	Stride	Output dimension	Activation	Mode
Input	1			(64,64,3)		Valid
Convolution	2	3 × 3	(1, 1)	(64,64,8)	RELU	
Max pooling	3	2 × 2		(32,32,8)		
Convolution	4	3 × 3		(32,32,8)		
Max pooling	5	2 × 2		(16,16,8)		
Dense	6			(10, 1)		
Dense	7			(2, 1)	Softmax	

**Table 2:** Size of dataset for three training strategy.

	SI-CNN	SD-CNN	ITSD-CNN
Training data	5760	648	648+transfer instance
Test data	720	72	72

**Table 3:** Classification accuracy of different training strategies.

Subjects	Accuracy % (mean ± std.dev.)		
	SI-CNN	SD-CNN	ITSD-CNN
1	82.3 ± 2.3	80.0 ± 2.9	93.2 ± 2.1
2	79.5 ± 5.1	76.7 ± 3.1	96.7 ± 3.2
3	63.8 ± 5.8	66.7 ± 3.2	94.1 ± 6.1
4	76.5 ± 3.2	83.3 ± 3.2	97.2 ± 1.0
5	79.8 ± 3.2	86.7 ± 2.9	92.7 ± 2.7
6	76.2 ± 4.7	79.0 ± 3.8	95.6 ± 2.5
7	77.6 ± 3.5	83.3 ± 2.1	94.9 ± 3.1
8	78.9 ± 2.8	83.3 ± 4.5	96.1 ± 0.8
9	81.3 ± 3.7	86.7 ± 3.5	92.2 ± 2.0
Ave	77.3 ± 3.8	80.6 ± 3.2	94.7 ± 2.6

**Table 4:** The review of classifiers performance for BCI competition IV dataset 2b.

Method	Researcher	Mean kappa value
FBCSP	Ang et al. [24]	0.502
Twin SVM	Soman and Jayadeva [25]	0.526
CNN-SAE	Tabar and Halici [23]	0.547
CSCNN	Rong et al. [26]	0.663
NCA + DTCWT	Malan and Sharma [27]	0.615
CNN-VAE	Dai et al. [17]	0.564
ITSD-CNN	Our method	0.664

## Data Availability

The data used to support the findings of this study are available from the corresponding author upon request.
